# Gene panel sequencing in heritable thoracic aortic disorders and related entities – results of comprehensive testing in a cohort of 264 patients

**DOI:** 10.1186/s13023-014-0221-6

**Published:** 2015-02-03

**Authors:** Laurence Campens, Bert Callewaert, Laura Muiño Mosquera, Marjolijn Renard, Sofie Symoens, Anne De Paepe, Paul Coucke, Julie De Backer

**Affiliations:** Center for Medical Genetics, Ghent University Hospital, De Pintelaan 185 - 0 K5, 9000 Ghent, Belgium; Cardiology Department, Ghent University Hospital, 9000 Ghent, Belgium

**Keywords:** Heritable Thoracic Aortic Disorders – next generation sequencing – Aneurysm, Dissecting/genetics – mutation detection rate

## Abstract

**Background:**

Heritable Thoracic Aortic Disorders (H-TAD) may present clinically as part of a syndromic entity or as an isolated (nonsyndromic) manifestation. About one dozen genes are now available for clinical molecular testing. Targeted single gene testing is hampered by significant clinical overlap between syndromic H-TAD entities and the absence of discriminating features in isolated cases. Therefore panel testing of multiple genes has now emerged as the preferred approach. So far, no data on mutation detection rate with this technique have been reported.

**Methods:**

We performed Next Generation Sequencing (NGS) based screening of the seven currently most prevalent H-TAD-associated genes (*FBN1*, *TGFBR1/2*, *TGFB2*, *SMAD3*, *ACTA2* and *COL3A1*) on 264 samples from unrelated probands referred for H-TAD and related entities. Patients fulfilling the criteria for Marfan syndrome (MFS) were only included if targeted *FBN1* sequencing and MLPA analysis were negative.

**Results:**

A mutation was identified in 34 patients (13%): 12 *FBN1*, one *TGFBR1*, two *TGFBR2*, three *TGFB2*, nine *SMAD3*, four *ACTA2* and three *COL3A1* mutations. We found mutations in *FBN1* (N = 3), *TGFBR2* (N = 1) and *COL3A1* (N = 2) in patients without characteristic clinical features of syndromal H-TAD. Six TAD patients harboring a mutation in *SMAD3* and one TAD patient with a *TGFB2* mutation fulfilled the diagnostic criteria for MFS.

**Conclusion:**

NGS based H-TAD panel testing efficiently reveals a mutation in 13% of patients. Our observations emphasize the clinical overlap between patients harboring mutations in syndromic and nonsyndromic H-TAD related genes as well as within syndromic H-TAD entities, justifying a widespread application of this technique.

**Electronic supplementary material:**

The online version of this article (doi:10.1186/s13023-014-0221-6) contains supplementary material, which is available to authorized users.

## Background

Thoracic Aortic Disorders (TAD), comprising aneurysms and dissections of the thoracic aorta (ascending, arch or descending part), are a prominent cause of morbidity and mortality entailing 0.5-1% of all-cause mortality in the Western World [1,2]. The etiology of TAD is complex and heterogeneous. Both genetic and acquired forms can be distinguished. The genetically determined forms can be categorized into syndromic and nonsyndromic heritable TAD (H-TAD), depending on the presence or absence of manifestations in other organ systems.

The prototype for syndromic H-TAD is Marfan syndrome (MFS), a multisystem disorder primarily affecting the ocular, skeletal and cardiovascular system. The main cause of morbidity and mortality in MFS is related to progressive dilatation and ensuing dissection of the aortic root. The diagnosis of MFS is based on the identification of clinical manifestations, as defined in the revised Ghent nosology, and may be supplemented with the identification of an underlying mutation in the *FBN1* gene, encoding the fibrillin-1 protein [3].

Over the last decade, many related syndromic H-TAD entities have been identified including Loeys-Dietz syndrome (LDS), caused by mutations in the genes encoding the TGFβ receptors 1 and 2 (*TGFBR1*/*2*). LDS is characterized in its most typical form by widespread arterial disease, arterial tortuosity, marfanoid features, hypertelorism and cleft palate/bifid uvula [4-6]. The clinical features of LDS show overlap, not only with MFS, but also with those of TGFβ-signaling related H-TAD entities [7], namely Aneurysm-Osteoarthritis syndrome (AOS), caused by *SMAD3* mutations, and H-TAD caused by mutations in the TGFβ2 ligand. *TGFB2* mutations were identified in several families displaying TAD in association with marfanoid features and clinically significant mitral valve prolapse [8-10]. In AOS, widespread arterial disease with frequent involvement of the visceral and iliac arteries is encountered. Osteoarthritis is a common additional feature [11-14]. Vascular Ehlers-Danlos (vEDS) syndrome, caused by mutations in the *COL3A1* gene, has been recognized as another important cause of widespread arterial disease. Patients with vEDS often present with arterial dissection without prior dilatation. Other characteristic clinical features include digestive tract and uterine ruptures, skin involvement (thin and translucent skin, severe easy bruising) and a distinctive facial appearance, which are at the basis of the Villefranche criteria [7,15-17].

Nonsyndromic H-TAD is genetically heterogeneous and practically all genes mentioned above have been reported in the context of this entity. Other associated genes include *ACTA2*, *MYH11*, *MYLK* and *PRKG1* [18-23], with *ACTA2* being the most prevalent one [24]. By definition, patients/families with nonsyndromic H-TAD will not present additional clinical manifestations although in some cases, other cardiovascular lesions, such as bicuspid aortic valve (BAV) and persistent ductus arteriosus (PDA) occur more frequently in *MYH11* and *ACTA2* related H-TAD. Mutations in the *ACTA2* gene trigger inappropriate proliferation of vascular smooth muscle cells leading to occlusive diseases such as coronary artery disease, stroke, Moyamoya disease and livedo reticularis. Additionally, iris floccule has been reported in *ACTA2* mutation carriers [18,19,25]. A very specific syndromic entity caused by the Arg179His *ACTA2* mutation is characterized by an early onset severe phenotype due to global smooth muscle cell dysfunction with PDA, congenital mydriasis, variable presentation of pulmonary hypertension, bladder and gastrointestinal problems and distinctive cerebrovascular features [26,27].

Often, the absence of gene-specific clinical manifestations and the large number of plausible genes hamper targeted gene testing in H-TAD, resulting in a time consuming and costly process. The need for high-throughput techniques enabling simultaneous testing of several genes was met by the progress made in the field of Next Generation Sequencing (NGS). Previously, our center reported a mutation analysis strategy using parallel sequencing of *FBN1* and *TGFBR1* and −*2* for the molecular diagnosis of MFS and LDS, respectively [28]. This method turned out to be time- and cost-effective compared to the conventional Sanger method [28]. In a next step, we implemented a novel molecular analysis strategy allowing simultaneous sequencing of seven H-TAD-associated genes‚ namely *FBN1*, *TGFBR1/2*, *TGFB2*, *SMAD3*, *ACTA2* and *COL3A1*.

Here we provide data on the mutation detection rate with gene panel screening in a large cohort of patients referred for genetic testing for H-TAD and related phenotypes. Based on these findings, we propose a practical guideline for clinical evaluation and genetic testing.

## Methods

Between November 2012 and April 2014, 264 consecutive samples of unrelated probands were sent to our center for H-TAD gene panel testing. Each patient was examined by his or her referring physician. For patients seen at the Center of Medical Genetics Ghent (CMGG) (N = 135), detailed phenotypic information was recorded in the patients’ electronic medical record. For patients referred from outside the CMGG, phenotypic information was provided by the referring physician (both geneticists and non-geneticists) via a check-list specific for H-TAD (Additional file 1), which included age, gender, suspected diagnosis, specifications regarding aortic/vascular disease and the option to indicate any observed phenotypic feature, including those listed in the revised Ghent nosology, and a pedigree [3]. Throughout the article patients presenting marfanoid features (systemic score ≥3^a^ and/or EL), LDS features (hypertelorism, bifid uvula and/or arterial tortuosity) and/or vascular EDS features (facial and/or skin features) are considered as syndromic patients. The term ‘distal aneurysms and dissections’ (DAD) refers to dilatation and/or dissection of arteries outside the thoracic aorta. A positive family history is defined as the presence of TAD in at least one 1st degree relative.

For patients fulfilling the revised Ghent criteria, targeted gene analysis is initiated, followed by MLPA if targeted gene analysis turns out negative. Panel testing is performed whenever overlapping and/or insufficient clinical features are present or when initial targeted gene testing and MLPA turns out negative in patients fulfilling the revised Ghent criteria.

### NGS

Seven genes associated with most commonly occurring and phenotypically overlapping syndromic and nonsyndromic H-TAD entities‚ were included in the panel; namely *FBN1*, *TGFBR1, TGFBR2, TGFB2*, *SMAD3*, *ACTA2* and *COL3A1*. Genes associated with a phenotype in which TAD is not the main characteristic, were not included (e.g. cutis laxa genes). New genes identified during the study period were not added to the panel in order to have a robust patient cohort in whom the same set of genes was tested. All coding regions and flanking sequences of each of the seven genes are PCR amplified in a fully automated fashion under identical reaction conditions. A high throughput PCR based approach was used for target enrichment. Primers were designed with PrimerXL (www.primerXL.org). In total 157 assays are used to cover the complete coding and splice site regions of the seven genes included in the panel.

PCRs are performed in 10 μl total volume. Uniform amplification conditions were applied. The PCR mixture consists out of 5 μl of KAPA2G Robust HotStart ReadyMix (2×, KAPABiosystems), 50 ng of DNA template and 2.5 μl of 1 μM forward en reverse primer (IDT). The temperature cycling protocol consists of initial denaturation step at 95°C for 3 min, followed by 35 cycles of denaturation at 95°C for 15 sec, annealing at 60°C for 10 sec, and an extension at 72°C for 15 sec. Final extension was accomplished at 72°C for 1 min. In a next step, all products are pooled and Next Generation Sequencing (NGS) using the Nextera protocol (Illumina) was performed on a MiSeq sequencer (Illumina). The sequence data was processed by a bioinformatic pipeline including the CLC bio Workbench 6.0.

Twelve samples were processed at the time. Minimal sequencing depth was set at 20× coverage. The overall mean coverage depth in our NGS approach was between 300x and 800x for all amplicons in the set of 7 genes. Three percent of the total number of exons in all patients scored below the targeted depth of 20×. These few amplicons (fragments differ from analysis to analysis), with incomplete coverage, were immediately analyzed with Sanger after the Miseq run [28]. This method enables a turn-around-time of approximately 8 weeks.

All mutations and unclassified variants found by NGS were confirmed on an independent DNA sample by Sanger sequencing.

NGS was completed for all genes in all cases, also when a mutation was identified.

### Pathogenicity assignment

The following information was obtained for each non-synonymous variant to determine pathogenicity: (1) the outcome of missense predictions tools (SIFT, PolyPhen2, Grantham Distance, Align GVGD, Mutation Taster), that are based on sequence homology, the physicochemical similarity between the alternate amino acids, effect of an amino acid substitution on the structure and function of a protein and conservation level of the amino acid residue amongst species, in case of mutations predicted to result in an amino acid substitution; (2) frequency of the variant in control alleles; (3) literature research including relevant phenotypic information, segregation studies and functional data. Variant segregation analysis was performed if DNA samples of affected and non-affected family members were available. For a variant possibly affecting a splice site the splicing effect at cDNA level was verified if cDNA of the patient was available.

Variants were considered as disease causing when there was convincing evidence from the above mentioned criteria about their pathogenicity. In most cases, a combination of different independent variant characteristics, supported the final decision.

When data remained inconclusive, the variant was classified as a variant of unknown significance (VUS). Data regarding the predicted pathogenicity of non-cysteine missense mutations in *FBN1*, of missense mutations in the other panel genes – not previously reported/provided by an rs number – and of VUS in *FBN1* are provided in Additional file 2.

### Statistics

The number of patients presenting a specific feature is given together with the number of patients in whom information regarding the particular feature was present (percentage in parentheses). χ^2^ and Fisher’s Exact tests were used to compare categorical variables. Data were analyzed with the unpaired sample t-test for normal-distributed continuous variables; non-normal distributed variables were compared using the Mann–Whitney-U test. Logistic regression analysis was applied to identify predictors for a positive genetic test result. A p-value of < 0.05 was used to define statistical significance (two-sided).

The study was approved by the ethical committee of the Ghent University Hospital.

## Results

Two hundred and sixty-four patients were included in the study (median age 42 years, IQR 28 – 52y). TAD was present in 233 patients (88%). In these TAD cases a positive family history for TAD was present in 27% (N = 62) and syndromic features were reported in 33% (N = 78).

Ten percent of cases (N = 24) had both a positive family history and syndromic features. The group of non-TAD patients (N = 31) had either (1) DAD (N = 23) – in 2 of them associated with a positive family history for TAD and in 3 others with syndromic features or (2) syndromic features without DAD (N = 8) – in four of them associated with a positive family history for TAD.

A causal mutation could be identified in 34 patients (13%): 12 *FBN1* (35.3%), one *TGFBR1* (2.9%), two *TGFBR2* (5.9%), three *TGFB2* (8.8%), nine *SMAD3* (26.5%), three *COL3A1* (8.8%) and four *ACTA2* (11.8%) mutations. Additionally, we found six VUS in *FBN1*. An overview of the different genotypes and clinical characteristics of mutation positive patients is provided in Tables 1 and 2, respectively.

**Table 1:** **Overview of mutations found in the different genes of the H-TAD panel and their associated phenotype**

	**ID**	**Age**	**MUTATIONS**		**Phenotype**	**Age**	**VUS**		**Phenotype**
			**Nucleotide change**	**Protein change**			**Nucleotide change**	**Protein change**	
*FBN1*	1	27	c.1879C > T [29]	p.(Arg627Cys)	S H-TAD	11	c.860A > G*	p.Glu287Gly	NS H-TAD
	2	1	c.3601 T > C*	p.(Cys1201Arg)	S H-TAD	16	c.2819G > T*	p.Gly940Val	S H-TAD
	3	3	c.4459 + 1G > C*	/	S H-TAD	56	c.3748G > T*	p.Asp1250Tyr	NS H-TAD
	4	54	c.5377 T > A*	p.(Cys1793Ser)	S H-TAD	32	c.4347G > T*	p.Glu1449Asp	NS H-TAD
	5	1	c.5558G > C*	p.(Cys1853Ser)	S H-TAD	47	c.7379A > G [30]	p.Lys2460Arg	NS H-TAD
	6	23	c.6496 + 1 G > A*	/	S H-TAD	39	c.7412C > G*	p.Pro2471Arg	NS H-TAD
	7	34	c.6952 T > C [29]	p.(Cys2318Arg)	NS H-TAD				
	8	52	c.7486 T > A*	p.(Cys2496Ser)	NS H-TAD				
	9	54	c.7486 T > A*	p.(Cys2496Ser)	NS H-TAD				
	10	40	c.7852G > A [31-33]	p.(Gly2618Arg)	TAD - SS a/o EL?				
	11	16	c.8536G > T*	p.(Glu2846X)	S H-TAD				
	12	8	c.8544dupA*	p.(Tyr2849IlefsX2)	S H-TAD				
*TGFBR1*	13	23	c.683_685delAAG [34]	p.(Glu228del)	S H-TAD				
*TGFBR2*	14	39	c.1084C > A*	p.(His362Asn)	S H-TAD				
	15	81	c.1379G > A [35]	p.(Arg460His)	NS DAD				
*TGFB2*	16	27	c.577C > T*	p.(Arg193Trp)	S H-TAD				
	17	48	c.980G > A [9]	p.(Arg327Gln)	S H-TAD				
	18	3	c.988C > T [10]	p.(Arg330Cys)	S H-TAD				
*SMAD3*	19	20	c.401-6G > A [36]	/	S H-TAD				
	20	53	c.546delT*	p.(Gly183AlafsX3)	S H-TAD				
	21	24	c.584_585insTC*	p.(Gln195HisfsX3)	S H-TAD				
	22	24	c.715G > A [37]	p.(Glu239Lys)	S H-TAD				
	23	53	c.715G > A [37]	p.(Glu239Lys)	S H-TAD				
	24	23	c.715G > A [37]	p.(Glu239Lys)	S H-TAD				
	25	14	c.859C > T [13]	p.(Arg287Trp)	S H-TAD				
	26	32	c.887 T > C*	p.(Leu296Pro)	S H-TAD				
	27	20	c.1155-2A > G*	/	S H-TAD				
*ACTA2*	28	61	c.115C > T [19,38]	p.(Arg39Cys)	NS H-TAD				
	29	17	c.455C > T [18]	p.(Arg149Cys)	NS H-TAD				
	30	59	c.772C > T [25]	p.(Arg258Cys)	NS H-TAD				
	31	32	c.910G > A [38]	p.(Gly304Ser)	NS H-TAD				
*COL3A1*	32	40	c.3410G > A [39]	p.(Gly1137Asp)	NS DAD				
	33	43	c.719G > T*	p.(Gly240Val)	NS H-TAD				
	34	35	c.811 C > T*	p.(Arg271X)	S-H-TAD				

(N)S H-TAD: (Non) Syndromic HTAD; NS DAD: Nonsyndromic distal aneurysms and dissections. SS a/o EL?: no info on the systemic score of Marfan syndrome and/or ophthalmological info available. *novel mutation, i.e. not previously reported in literature and not previously found in our lab. Age is expressed in years.

**Table 2:** **Overview clinical features per genotype**

	**MFS features**				**LDS features**	**vEDS**	**TAD**	**MVP**	**DAD**	**FH TAD**
	**SS**			**EL**						
	**SS1-2**	**SS3-6**	**SS ≥ 7**							
FBN1 (N = 12)	/	5/11	3/11	-	1	1	10	3	-	4/8
TGFBR1 (N = 1)	+	/	/	-	+	-	+	-	-	-
TGFBR2 (N = 2)	1	/	/	-	1	-	1	-	1	1
TGFB2 (N = 3)	/	2	1	-	-	-	3	1	1	2
SMAD3 (N = 9)	1	2	6	-	1	-	8	3	-	6/8
ACTA2 (N = 4)	/	/	/		-	-	4	-	1	2/3
COL3A1 (N = 3)	-				-	1	2	-	2	-

DAD: distal aneurysms a/o dissections; EL: ectopia lentis; FH TAD: positive family history for thoracic aneurysms and dissections; MVP: mitral valve prolapse; SS: systemic score; TAD: thoracic aneurysms and dissections. MFS (Marfan syndrome) features i.e. systemic features and/or ectopia lentis, LDS (Loeys-Dietz syndrome) features i.e. hypertelorism, bifid uvula and/or arterial tortuosity, vEDS (Ehlers-Danlos syndrome) features i.e. facial and/or skin features.

The number of patients of whom clinical data was provided is given whenever data regarding a specific features was not available for all patients.

Thoracic aortic disease was present in 29 out of the 34 mutation carriers (85%), in whom a positive family history for TAD was present in 45% (N = 13), syndromic features were reported in 69% (N = 20), 28% of patients (N = 8) had both a positive family history and syndromic features and 17% of patients (N = 5) had no known family history for TAD and no syndromic features. The five remaining patients demonstrated DAD (N = 2 – one with positive family history for TAD) or syndromic features (N = 3 – one with positive family history for TAD).

In Table 3, we compared the demographic and clinical characteristics in patients with and without a mutation in the seven H-TAD genes (in Additional file 3 children are presented separately from adult cases). There was a male preponderance in the study cohort, presumably due to an up to two times higher prevalence of TAD in males compared to females and to a known bias in the diagnosis of cardiovascular disease in woman in general [1,40-42]. A positive family history, age and presence of syndromic features (as specified in the method section), were identified as the strongest predictors for a positive genetic test result using logistic regression analysis (p = 0.001 to 0.01). The regression model, in which age, family history and presence of syndromic features were included, indicated an approximately three times higher chance of finding a mutation when the family history for TAD was positive or when syndromic features were present (p = 0.033 and 0.035 – Additional file 4). Age was no significant independent predictor in this model (p = 0.092 – Additional file 4).

**Table 3:** **Main clinical characteristics of mutation negative and mutation positive patients**

	**Mutation negative** ^**a**^ **(N = 224)**	**Mutation positive (N = 34)**	**p-value**
Age (years)	41.8 (17.0)	31.9 (19.5)	0.004
Female gender	63/224	17/34	0.01
TAD	199/224	29/34	0.55
DAD	52/224	5/34	0.27
Family history for TAD	52/179	15/28	0.01
Systemic score			
1-2	47/164	3/33	0.018
3-6	45/164	9/33	0.98
≥7	13/164	10/33	<0.001
Mitral valve prolapse	23/216	7/34	0.097
Bifid uvula	2/139	2/15	0.048
Hypertelorism	5/224	3/34	0.039
Arterial tortuosity	12/77	0/7	0.26
Skin a/o facial features vEDS	15/142	2/19	0.99

^a^Patients in whom a VUS was identified are not included in Table 3.

DAD: distal aneurysms a/o dissections; TAD: thoracic aortic disease; vEDS: vascular Ehlers-Danlos syndrome. Age is presented as mean with standard deviation in parentheses, the number of patients positive for the respective feature and the number of patients of whom clinical data was provided, is given.

Of the 12 patients harboring an *FBN1* mutation, eight had a clinical suspicion for MFS prior to the molecular test, but did not fulfil the revised Ghent criteria (N = 6) or demonstrated overlapping features with other syndromic entities (N = 2) (Table 2). The four remaining *FBN1* patients all presented TAD, three of them demonstrated no syndromic features (systemic score of 0 – no EL); in one patient no information on the MFS systemic score and/or ophthalmological findings was provided.

Nineteen patients, in whom no mutation in *FBN1* could be identified, fulfilled the revised Ghent criteria. Subsequent panel testing revealed mutations in *SMAD3* (N = 6) and in *TGFB2* (N = 1). The remaining patients with a *SMAD3* (N = 3) and with a *TGFB2* (N = 2) mutation demonstrated some marfanoid features (Table 2).

One *ACTA2* mutation carrier demonstrated coronary artery disease, but other features associated with *ACTA2* mutations [24] were either not present or not reported. None of the patients with a *TGFBR1* or *2* mutation demonstrated the classic LDS trias (hypertelorism, cleft palate/bifid uvula and aortic/arterial aneurysms/tortuosity), but two of them did present some particular LDS features such as hypertelorism or bifid uvula. Surprisingly, two out of the three patients with a *COL3A1* mutation did not present skin or facial features reminiscent of vascular Ehlers-Danlos Syndrome (vEDS). One of them, previously diagnosed with a type A aortic dissection at the age of 31, presented with a spontaneous rupture of an intercostal artery. The second vEDS patient, who did not fulfil the Villefranche criteria, presented at 40 years of age with simultaneous dissections of several visceral side branches of the aorta (superior mesenterial artery, truncus coeliacus and right common iliacal artery). His family history was highly suspicious for a heritable trait as both his mother and sister had died suddenly of an arterial dissection outside the aorta. The third *COL3A1* patient, carrying a null mutation (Table 1), did present facial vEDS characteristics. He presented a type B aortic dissection at 35 years of age. His brother experienced a coronary artery dissection at 37 years of age.

In the patients in whom a VUS in *FBN1* was identified, TAD was reported in all but one (Table 1). This particular patient, 16 years of age, was the only patient with a VUS who demonstrated skeletal features reminiscent of MFS. Pathogenicity data of all VUS are provided in Additional file 2.

Eight patients referred for panel testing had isolated intracerebral aneurysm/dissection or dissection of the neck arteries without syndromic features and a negative family history for TAD. Panel testing was negative in all of them.

In total, 34 patients in the study cohort had a bicuspid aortic valve (BAV), all but one with associated ascending aortic dilatation. In six patients a positive family history and in six syndromic features were reported. Panel testing was negative in all BAV patients.

## Discussion and conclusions

The identification of a causal mutation underlying H-TAD is important with regard to management and the evaluation of relatives at risk. We report on the results of NGS-based panel testing of the 7 most frequently reported H-TAD genes in a cohort of 264 unrelated individuals.

The primary purpose of our study was to determine the mutation detection rate in a representative sample of patients referred to a large genetic center for clinical genetic testing for H-TAD and related disorders. The overall mutation detection rate in the total cohort and in the subgroup of H-TAD patients was 13 and 12%, respectively.

The majority of mutation positive patients harbored mutations in *FBN1* (35.3%) and *SMAD3* (26.5%). To the best of our knowledge this is the first time that such a high mutation detection rate for *SMAD3* in an H-TAD cohort is reported. The patients in whom an *FBN1* mutation was identified by panel testing (N = 12), did not fulfil the revised Ghent criteria (prior to molecular testing) or demonstrated overlapping features with other H-TAD related entities. Panel sequencing allowed us to establish the diagnosis of MFS – following the revised Ghent criteria – in 10 of them. Targeted *FBN1* gene analysis is in general initiated in those patients who clearly fulfill the diagnostic criteria for MFS (prior to molecular testing) and who do not present specific clinical features triggering another syndromic diagnosis, because of a high pretest probability of finding an *FBN1* mutation in this particular population [28]. If targeted screening and MLPA turn out negative; NGS is initiated. Since the introduction of the H-TAD panel in our lab and thus start of inclusion for the current study 57 mutations have been identified with targeted *FBN1* analysis and three with MLPA (not included in the current study). This implicates that mutations in this gene remain the most frequent cause of syndromic H-TAD and further supports targeted gene analysis in clear cases of MFS. Remarkably, patients fulfilling the revised Ghent criteria may also harbor mutations in other H-TAD genes, as evidenced by a notable number of *SMAD3* mutations (N = 6) and one *TGFB2* mutation on a total of 19 *FBN1* negative patients. These findings emphasize the clinical overlap between syndromic H-TAD entities and warrant NGS panel testing in patients negative for initial targeted gene analysis.

Patients presenting with nonsyndromic H-TAD (N = 155) may harbor mutations in any of the known H-TAD genes. In addition to previously reported *FBN1, TGFBR1/2*, *TGFB2*, *SMAD3* and *ACTA2* mutations [9,12,18,19,24,35,43], we report on a *COL3A1* mutation in a nonsyndromic H-TAD patient. The mutation detection rate in the *FBN1* gene in the nonsyndromic H-TAD patient group (1.9%) is in line with previously published data [44]. The mutation detection rate in *ACTA2* (2.6%) contrasts with what has been reported in the first studies identifying *ACTA2* mutations in up to 16% of nonsyndromic H-TAD families [18-20]. This discrepancy may be due to differences in study design and patient ascertainment. Our results are in line with more recent studies performed in similar patient populations [44].

In the group of non-TAD patients presenting DAD at the time of diagnosis (N = 21) we found one *COL3A1* and one *TGFBR2* mutation. The finding of *COL3A1* mutations in patients without any facial or skin features evocative for vEDS is quite remarkable and warrants a high index of suspicion for the disease among treating physicians.

In patients presenting syndromic features in the absence of TAD and DAD at the time of diagnosis (N = 8), two *FBN1* and one *SMAD3* mutation were found. These were all young patients (age range 16–23 years), in whom aortic disease had not yet developed.

Most of the *FBN1* mutations found in our cohort were substitutions of a highly conserved cysteine residue involving a cb-EGF-like domain, which is the most common pathogenic *FBN1* mutation type [29]. Two intronic mutations at the consensus splice site residues +1 were identified and four patients demonstrated a mutation leading to a premature termination codon. In three patients a substitution of a non-cysteine amino acid was found. Most *COL3A1* mutations are glycine substitutions leading to disruption of the triple helix structure of type III collagen [7], and were identified in two of our patients. In a minority of vEDS patients *COL3A1* null mutations leading to haploinsufficiency are identified [45]. Those patients generally present with a milder clinical phenotype with delayed onset of arterial complications, less visceral complications and less commonly characteristic skin and/or facial features [46-48]. The case we describe here, demonstrates early onset arterial disease with some facial features reminiscent of vEDS, but without other major and minor diagnostic criteria for vEDS [17].

In most cases, interpretation of the functional consequence of typical mutations in *FBN1* (cysteine missense mutations) and in *COL3A1* (glycine missense mutations) is clear-cut. However, a significant proportion of variants, named VUS, consists of ‘atypical’ missense changes or potential splice-site changes of uncertain biologic or clinical relevance. The challenging issue remains how to use these data in clinical practice.

In the current era of high-throughput molecular genetic techniques, knowledge of the indications and limitations for these tests in daily clinical practice is increasingly important. Pending guidelines, supported by an international expert panel, we suggest the following recommendations for clinical evaluation and for guiding clinicians in their decision to refer patients, presenting with TAD and/or DAD, for panel testing:

- Genetic counseling is recommended for all patients with TAD when their disease cannot be explained solely by non-genetic causes
- It is recommended to clinically investigate first degree relatives (siblings – parents – offspring) of a subject with TAD to identify familial forms [49,50]
- Panel testing is recommended in patients presenting TAD/DAD and with a positive family history of TAD, to investigate the molecular cause underlying the H-TAD
- In patients fulfilling the revised Ghent criteria, targeted *FBN1* analysis can be considered prior to panel testing
- In patients without TAD presenting syndromic features suggestive of H-TAD ^b^ and a positive family history of TAD, panel testing is recommended ^c^, to investigate the molecular cause underlying the H-TAD
- In isolated (non-familial), non-syndromic TAD, panel testing can be considered if the disease is not due to a high cardiovascular risk profile ^d^ and if genetic testing of their relatives is relevant
- In isolated (non-familial), non-syndromic DAD (with the exclusion of single aneurysms/dissections of head/neck arteries and abdominal aorta ^e^), panel testing can be considered if the disease is not due to a high cardiovascular risk profile ^d^ and if genetic testing of their relatives is relevant
- It is recommended that genetic testing is performed in certified diagnostic laboratories with expertise in interpretation of H-TAD-related mutations
- In patients with non-syndromic TAD associated with BAV in the absence of a positive family history, panel testing is not routinely recommended
- In young patients (<30 years), presenting syndromic features ^b^ (not fulfilling the revised Ghent criteria), but no TAD/DAD and no family history of TAD, the threshold for clinical follow-up should in general be kept low.

### Conclusion

It is clear that the development and implementation of these new technologies in the diagnostics of H-TAD leads to a more time- and cost-efficient strategy to identify disease causing mutations. The turnaround time for the NGS panel is approximately 8 weeks, which is clearly shorter than the conventional method with Sanger (~12 weeks/gene), and the reagent cost per amplicon is about 4 times lower with NGS. In selected cases with a high pre-test probability of an *FBN1* mutation, for instance patients fulfilling the revised Ghent criteria including the presence of ectopia lentis, targeted gene testing is still justified. At least, we recommend panel testing in those cases where the phenotype is not conclusive or when initial targeted gene analysis did not reveal a mutation.

### Limitations

The NGS technology fails to detect larger deletions or insertions. Therefore, mutation-negative samples of patients with a high clinical suspicion should still be evaluated by a complementary technique, such as MLPA [28]. MLPA of *FBN1* was performed prior to inclusion in the study in patients fulfilling the revised Ghent criteria who were negative for targeted *FBN1* analysis. MLPA of the other panel genes was not performed. Therefore the number of mutation positive patients could be slightly underestimated.

It is not excluded that mutation negative patients carry a pathogenic mutation in a gene‚ not included in the current panel. The mutation detection rate presented here could therefore be somewhat underestimated. Sequencing of a second gene panel, comprising the more rare H-TAD genes and genes that are associated with a phenotype, in which TAD is not a primary feature, is ongoing. Our diagnostic panel has been expanded now to include *MYH11*, *TGFB3*, *PRKG1* and *MLCK*.

### Consent

Informed consent was obtained from all patients and the Declaration of Helsinki protocols were followed.

## Endnotes

^a^A systemic score of 3 or more was considered as a marfanoid phenotype.

^b^Syndromic features MFS (skeletal features – EL) – LDS features (hypertelorism, bifid uvula a/o arterial tortuosity) – vEDS features (skin a/o facial vEDS features).

^c^Young patients (<30 years) may not yet present TAD, therefore the threshold for genetic testing should remain low whenever a suspicious family history or convincing and specific syndromic features - such as EL - are present.

^d^High cardiovascular risk: untreated arterial hypertension + ≥1 CV risk factor OR ≥3 CV risk factors (CV risk factors: BMI > 30 kg/m^2^, diabetes, arterial hypertension, hyperlipidemia, smoking and positive family history for coronary artery disease).

^e^Isolated aneurysm/dissection of the abdominal aorta or an intracranial or neck artery is less suspicious for H-TAD than aneurysm/atraumatic dissection of middle and small sized thoracic and/or abdominal arteries.

## Additional files

Additional file 1:
**Clinical check-list specific for H-TAD.**
Additional file 2:
**Pathogenicity data of missense mutations and VUS.** Data regarding the predicted pathogenicity of non-cysteine missense mutations in *FBN1*, of missense mutations in the other panel genes – not previously reported/provided by an rs number – and of VUS in *FBN1*. Additional file 3:
**Main clinical characteristics of mutation negative and mutation positive patients, presented separately for children ≤15 years and adults.**
Additional file 4:
**Logistic regression analysis with ‘positive genetic test result’ as outcome variable.**
