# Supplementary material for: Gene panel sequencing in heritable thoracic aortic disorders and related entities – results of comprehensive testing in a cohort of 264 patients
Source: Orphanet J Rare Dis. 2015 Feb 3;10:9. doi: 10.1186/s13023-014-0221-6 (PMC4326194; doi:10.1186/s13023-014-0221-6)
Supplement: Additional file 1: — Clinical check-list specific for H-TAD. [file 13023_2014_221_MOESM1_ESM.doc]

**CLINICAL INFORMATION SHEET**

**Marfan syndrome and related aortic aneurysm syndromes**

**Patient information**

Name:

First Name(s):

| Sex: | M | F |  |
| --- | --- | --- | --- |

Date of Birth (dd/mm/yyyy):     /     /

Address:

Referring Physician:

Referring Center:

| SAMPLE: | EDTA blood | DNA | Skin biopsy | Chorionic villi |
| --- | --- | --- | --- | --- |
|  | Heparin blood | RNA | Aortic biopsy | Amniocytes |
|  | Buccal swab | Fibroblasts | Paraffin embedded material | |
|  | Other: | | | |

**Date** (dd/mm/yyyy):      /     /

**Sample arrived:**

**Suspected diagnosis**

Marfan syndrome

Ehlers-Danlos syndrome

Loeys-Dietz syndrome

Shprintzen-Goldberg syndrome

Beals-Hecht syndrome or congenital contractural arachnodactyly

Arterial tortuosity syndrome

Heritable (thoracic) aortic aneurysms syndrome (syndromal and non-syndromal)

Bicuspid aortic valve

Other:

This checklist is meant to guide genetic testing for the Marfan syndrome or testing in the setting of (familial) thoracic aortic aneurysm/dissection and/or arterial tortuosity. Since both entities are not mutually exclusive, both check-lists may be used for a single patient in some cases.

Selecting the most likely gene to be screened in order to explain the underlying clinical presentation in your patient highly depends on adequate and correct clinical data. We therefore kindly ask you to be as precise and specific as possible.

The differential diagnosis in patients referred for additional genetic testing with a clinical presentation characterized by aortic (root) aneurysm/dissection and/or arterial tortuosity is extensive. You will find an overview of possible diagnosis below. Please indicate what diagnosis you suspect in your patient and/or make sure to fill out the checklist as complete as possible so that we can set up the appropriate genetic testing.

**CLINICAL SUMMARY**

|  |
| --- |

**PEDIGREE**

|  |
| --- |

| Differential diagnosis | Gene | Discriminating features |
| --- | --- | --- |
| Marfan syndrome (MFS) | FBN1 | Aortic root dilatation, presence of ectopia lentis, systemic features (table 1) (diagnostic criteria Box 1) |
| Loeys-Dietz syndrome (LDS) | TGBR1/2  TGFB2  SMAD3 | Bivid uvula/cleft palate, arterial tortuosity, hypertelorism, diffuse aortic and arterial aneurysms, craniosynostosis |
| Shprintzen-Goldberg syndrome (SGS) | SKI (FBN1) | Mild aortic root dilatation, mitral valve prolapse, craniosynostosis, mild-to-modertate intellectual disability |
| Congenital contractural arachnodactyly (CCA) | FBN2 | Crumpled ears, contractures |
| Weill-Marchesani syndrome (WMS) | FBN1 and ADAMTS 10 | Microspherophakia, brachydactyly, joint stiffness, short stature |
| Ectopia lentis syndrome (ELS) | FBN1, LTBP2,  ADAMTS4 | Lack of aortic root dilatation |
| Homocystinuria | CBS | Thrombosis, mental retardation |
| Heritable Thoracic Aortic aneurysm/Dissection (H-TAD) (syndromal and non-syndromal) | TGFBR1/2 | Lack of Marfanoid skeletal features, |
|  | ACTA2 | Livedo reticularis, iris flocculi, CVA |
|  | SMAD3 | Osteoarthritis, arterial tortuosity, arterial aneurysms and dissections, intracranial aneurysms |
|  | TGFB2 | Mitral Valve Prolapse, cerebrovascular disease, arterial tortuosity |
|  | MLCK | Gastro-intestinal abnormalities |
|  | PRKGA1 | Arterial aneurysms and dissections, arterial tortuosity |
| H-TAD with bicuspid aortic valve (BAV) | ACTA2 | BAV, lack of Marfanoid skeletal features, levido reticularis, iris flocculi |
|  | SMAD6 | BAV, lack of Marfanoid skeletal features, coarctatio aortae |
| FH-TAD with patent ductus arteriosus (PDA) | MYH11 | PDA, lack of Marfanoid skeletal features |
| Arterial tortuosity syndrome (ATS) | SLC2A10 | Generalised arterial toruosity, arterial stenosis, facial dysmorphism |
| Ehlers-Danlos syndromes (vascular, valvular) | COL3A1, COL1A2, | Middle sized artery aneurysm, severe valvular insufficiency, translucent skin, dystrophic scars, facial characteristics |
| Cutis laxa | ELN (AD) | Cutis laxa with variable involvement of internal organs (lung, aorta), association with BAV |
|  | FBLN4 (AR) | Cutis laxa, emphysema, arterial tortuosity, aortic aneurysm, joint laxity, pectus excavatum, diaphragmatic hernia, bone fragility |

**Check-list for Heritable Thoracic Aortic Aneurysm/Dissection and/or arterial tortuosity**

| Suspected clinical diagnosis (see list above): | |
| --- | --- |
| Maximal aortic diameter | mm |
| Age at measurement | yrs |
| Localisation of the maximum dilatation |  |
| Sinus Valsalva | <INPUT TYPE=\ |
| Sinotubular Junction | <INPUT TYPE=\ |
| Ascending Aorta | <INPUT TYPE=\ |
| Aortic arch | <INPUT TYPE=\ |
| Descending Aorta | <INPUT TYPE=\ |
| Abdominal Aorta | <INPUT TYPE=\ |
| Aortic dissection: | |
| thoracic type A – type B | A -B |
| abdominal | <INPUT TYPE=\ |
| Arterial tortuosity | <INPUT TYPE=\ |
| Peripheral arterial dissection: please specify | |
| Bicuspid Aortic Valve | <INPUT TYPE=\ |
| Other cardiovascular lesions (please specify): | |
| Other systemic features (please specify):   - Ocular: - Osteo-articular: - Central Nervous: - Skin: - Gastro-intestinal: | |

**Revised Ghent Criteria for Diagnosis of Marfan syndrome and related conditions**

**In the absence of family history**:

(1) Ao (Z≥2) + EL = MFS

(2) Ao (Z≥2) + FBN1 = MFS

(3) Ao (Z≥2) + Syst (≥7pts) = MFS

(4) EL + FBN1 with known Ao = MFS

**In the presence of family history**:

(5) EL + FH of MFS (as defined above) = MFS

(6) Syst (≥7 pts) + FH of MFS (as defined above) = MFS

(7) Ao (Z≥2 in adults, Z≥3 in children) + FH of MFS (as defined above) = MFS

Z: Z-score (aortic root diameter corrected for age and BSA); EL: ectopia lentis; FBN1: Fibrillin 1 mutation; Syst: systemic score (see below); FBN1 with known Ao: FBN1 mutation linked to aortic aneurysm in other patients/families (Loeys et al, Journal of Medical Genetics 2010)

Required clinical data

| Aortic diameter at the level of the sinus of Valsalva | mm |
| --- | --- |
| Age at measurement | yrs |
| Height | cm |
| Weight | kg |
| Ectopia Lentis | Y/N |
| Systemic score | /20 |
| Family History: please specify | |

NE = not examined

| Systemic features | Yes | No | NE | Score |
| --- | --- | --- | --- | --- |
| Wrist AND thumb sign | <INPUT TYPE=\ | <INPUT TYPE=\ | <INPUT TYPE=\ | 3 |
| Wrist OR thumb sign | <INPUT TYPE=\ | <INPUT TYPE=\ | <INPUT TYPE=\ | 1 |
| Pectus carinatum deformity | <INPUT TYPE=\ | <INPUT TYPE=\ | <INPUT TYPE=\ | 2 |
| Pectus excavatum or chest asymmetry | <INPUT TYPE=\ | <INPUT TYPE=\ | <INPUT TYPE=\ | 1 |
| Hindfoot deformity | <INPUT TYPE=\ | <INPUT TYPE=\ | <INPUT TYPE=\ | 2 |
| Pes Planus | <INPUT TYPE=\ | <INPUT TYPE=\ | <INPUT TYPE=\ | 1 |
| Pneumothorax | <INPUT TYPE=\ | <INPUT TYPE=\ | <INPUT TYPE=\ | 2 |
| Dural ectasia | <INPUT TYPE=\ | <INPUT TYPE=\ | <INPUT TYPE=\ | 2 |
| Protrusio acetabuli | <INPUT TYPE=\ | <INPUT TYPE=\ | <INPUT TYPE=\ | 2 |
| Reduced US/LS AND increased arm/height AND no severe scoliosis | <INPUT TYPE=\ | <INPUT TYPE=\ | <INPUT TYPE=\ | 1 |
| Scoliosis or thoracolumbar kyphosis | <INPUT TYPE=\ | <INPUT TYPE=\ | <INPUT TYPE=\ | 1 |
| Reduced elbow extension | <INPUT TYPE=\ | <INPUT TYPE=\ | <INPUT TYPE=\ | 1 |
| Facial features (3/5) (dolichocephaly, enophthalmos,  downslanting palpebral fissures, malar hypoplasia, retrognathia) | <INPUT TYPE=\ | <INPUT TYPE=\ | <INPUT TYPE=\ | 1 |
| Skin striae | <INPUT TYPE=\ | <INPUT TYPE=\ | <INPUT TYPE=\ | 1 |
| Myopia > 3 diopters | <INPUT TYPE=\ | <INPUT TYPE=\ | <INPUT TYPE=\ | 1 |
| Mitral valve prolapse (all types) | <INPUT TYPE=\ | <INPUT TYPE=\ | <INPUT TYPE=\ | 1 |
| **TOTAL SCORE** | **/20** | | | |

Maximum total: 20 points; score> 7 indicates systemic involvement
