# Supplementary material for: Gene panel sequencing in heritable thoracic aortic disorders and related entities – results of comprehensive testing in a cohort of 264 patients
Source: Orphanet J Rare Dis. 2015 Feb 3;10:9. doi: 10.1186/s13023-014-0221-6 (PMC4326194; doi:10.1186/s13023-014-0221-6)
Supplement: Additional file 2: — Pathogenicity data of missense mutations and VUS. Data regarding the predicted pathogenicity of non-cysteine missense mutations in FBN1, of missense mutations in the other panel genes – not previously reported/provided by an rs number – and of VUS in FBN1. [file 13023_2014_221_MOESM2_ESM.docx]

Pathogenicity data of missense mutations* and VUS

|  | Gene | Protein level | Protein domain | Conservation within cbEGF domain (*FBN1*) | Grantham Distance | In silico prediction of effect | | | Rs number | Frequency  Population studies | Literature | Segregation analysis |
| --- | --- | --- | --- | --- | --- | --- | --- | --- | --- | --- | --- | --- |
|  |  |  |  |  |  | SIFT | Polyphen2 | Mutation taster |  |  |  |  |
| MUTATIONS | FBN1 | p.Arg627Cys ^a^ | cb-EGF | - Arg poorly conserved within cbEGF | Large (180) | Deleterious | Probably damaging | Disease causing | / | / | ^1^ D  Previously found at CMGG | de novo mutation |
|  |  |  |  |  |  |  |  |  |  |  |  |  |
|  |  |  |  |  |  |  |  |  |  |  |  |  |
|  |  | p.Gly2618Arg | cb-EGF | - Gly poorly conserved within cbEGF | Moderate (125) | Deleterious | Probably damaging | Disease causing | rs141133182 | ESP: EA 1/8591 – AA 1/4395 – total 2/12986 | ^2-4^ D  Previously found at CMGG | NA |
|  | TGFBR2 | p.His362Asn | Serine threonine kinase domain |  | Small (68) | Deleterious | Probably damaging | Disease causing | / | / | / | NA |
|  |  |  |  |  |  |  |  |  |  |  |  |  |
|  | TGFB2 | p.Arg193Trp | LAP |  | Moderate (101) | Deleterious | Probably damaging | Disease causing | / | / | / | segregates |
|  | SMAD3 | p.Leu296Pro | MH2 domain |  | Moderate (98) | Deleterious | Probably damaging | Disease causing | / | / | / | Segregates |
| VUS | FBN1 | p.Glu287Gly ^b^ | cb-EGF | - Glu poorly conserved within cbEGF | Moderate (98) | Deleterious | Possible damaging | Disease causing | / | / | / | NA |
|  |  | p.Gly940Val ^b^ | cb-EGF | - Gly highly conserved within cbEGF | Moderate (109) | Deleterious | Probably damaging | Disease causing | / | / | / | NA |
|  |  | p.Asp1250Tyr | cb-EGF | - Asp poorly conserved within cbEGF | Large (160) | Deleterious | Probably damaging | Disease causing | / | / | / | inconclusive |
|  |  | p.Glu1449Asp | cb-EGF | - Glu highly conserved within cbEGF | Small (45) | Deleterious | Probably damaging | / | / | / | / | NA |
|  |  | p.Lys2460Arg | cb-EGF | - Lys poorly conserved within cbEGF | Small (26) | Deleterious | Probably damaging | Disease causing | rs144189837 | ESP: EA 2/8590 – AA 0/4396 – total 2/12986 | ^5^ VUS | NA |
|  |  | p.Pro2471Arg | cb-EGF | - Pro poorly/moderately conserved within cbEGF | Moderate (103) | Deleterious | Probably damaging | / | / | / | / | NA |

*For *FBN1* only non-cysteine missense mutations are presented, for the other panel genes data of missense mutations not previously reported/provided by an rs number are given.

SIFT: <https://sift.jvci.org>; Polyphen 2: <http://genetics.bwh.harvard.edu/pph2/>. D: disease causing; NA: not available;

^a^ de novo mutation

^b^ possibly affects splicing, however the functional effect of the variant could not be analyzed, because cDNA was not available upon request.

Reference List

1. Collod-Beroud,G, Le, BS, Ades, L, Ala-Kokko, L, Booms, P, Boxer, M, Child, A, Comeglio, P, De Paepe, A, Hyland, JC, Holman, K, Kaitila, I, Loeys, B, Matyas, G, Nuytinck, L, Peltonen, L, Rantamaki, T, Robinson, P, Steinmann, B, Junien, C, Beroud, C, and Boileau, C. Update of the UMD-FBN1 mutation database and creation of an FBN1 polymorphism database. Hum. Mutat. 2013,22:199-208.

2. Loeys,B, Nuytinck, L, Delvaux, I, De Bie, S, and De Paepe, A. Genotype and phenotype analysis of 171 patients referred for molecular study of the fibrillin-1 gene FBN1 because of suspected Marfan syndrome. Arch. Intern. Med. 2001,161:2447-2454.

3. Comeglio,P, Johnson, P, Arno, G, Brice, G, Evans, A, Aragon-Martin, J, da Silva, FP, Kiotsekoglou, A, and Child, A. The importance of mutation detection in Marfan syndrome and Marfan-related disorders: report of 193 FBN1 mutations. Hum. Mutat.2007,28:928.

4. Turner,CL, Emery, H, Collins, AL, Howarth, RJ, Yearwood, CM, Cross, E, Duncan, PJ, Bunyan, DJ, Harvey, JF, and Foulds, NC. Detection of 53 FBN1 mutations (41 novel and 12 recurrent) and genotype-phenotype correlations in 113 unrelated probands referred with Marfan syndrome, or a related fibrillinopathy. Am. J. Med. Genet. 2009,149A:161-170.

5. Robinson,DO, Lin, F, Lyon, M, Raponi, M, Cross, E, White, HE, Cox, H, Clayton-Smith, J, and Baralle, D. Systematic screening of FBN1 gene unclassified missense variants for splice abnormalities. Clin. Genet. 2012,82:223-231.
