# Supplementary material for: Gene panel sequencing in heritable thoracic aortic disorders and related entities – results of comprehensive testing in a cohort of 264 patients
Source: Orphanet J Rare Dis. 2015 Feb 3;10:9. doi: 10.1186/s13023-014-0221-6 (PMC4326194; doi:10.1186/s13023-014-0221-6)
Supplement: Additional file 3: — Main clinical characteristics of mutation negative and mutation positive patients, presented separately for children ≤15 years and adults. [file 13023_2014_221_MOESM3_ESM.docx]

Main clinical characteristics of mutation negative and mutation positive patients, data are presented separately for children ≤15 years and adults.

|  | Mutation negative^a^ | | Mutation positive | | |
| --- | --- | --- | --- | --- | --- |
|  | ≤15  (N=22) | >15  (N=202) | ≤15  (N=6) | | >15  (N=28) |
| Female gender | 7/22 | 56/202 | 4/6 | 13/28* | |
| TAD | 21/22 | 178/202 | 6/6 | 23/28 | |
| DAD | 2/22 | 50/202 | 0/6 | 5/28 | |
| Family history for TAD | 3/13 | 49/166 | 1/3 | 14/25* | |
| Systemic score |  | |  | | |
| 1-2 | 3/18 | 44/146 | 1/6 | 2/27* | |
| 3-6 | 6/18 | 39/146 | 5/6 | 4/27 | |
| ≥ 7 | 3/18 | 10/146 | 0/6 | 10/27† | |
| Mitral valve prolapse | 1/21 | 22/195 | 3/6* | 4/28 | |
| Bifid uvula | 0/11 | 2/128 | 0/3 | 2/12* | |
| Hypertelorism | 2/22 | 3/202 | 1/6 | 2/28 | |
| Arterial tortuosity | 0/3 | 12/74 | 0/1 | 0/6 | |
| Skin a/o facial features vEDS | 0/11 | 15/131 | 0/2 | 2/17 | |

^a^Patients in whom a VUS was identified are not included in table 3.

DAD: distal aneurysms a/o dissections; TAD: thoracic aortic disease; vEDS: vascular Ehlers-Danlos syndrome. Age is presented as mean with standard deviation in parentheses and the number of patients positive for the respective feature and the number of patients of whom clinical data was provided, is given. † p-value below 0.001; *p-value below 0.05 (for comparison of mutation positive and negative patients)
