# Supplementary material for: Gene panel sequencing in heritable thoracic aortic disorders and related entities – results of comprehensive testing in a cohort of 264 patients
Source: Orphanet J Rare Dis. 2015 Feb 3;10:9. doi: 10.1186/s13023-014-0221-6 (PMC4326194; doi:10.1186/s13023-014-0221-6)
Supplement: Additional file 4: — Logistic regression analysis with ‘positive genetic test result’ as outcome variable. [file 13023_2014_221_MOESM4_ESM.docx]

Logistic regression analysis with ‘positive genetic test result’ as outcome variable.

|  | | B | SE | P value | Exp(B) | 95% C.I.for Exp(B) | |
| --- | --- | --- | --- | --- | --- | --- | --- |
|  |  |  |  |  |  | Lower | Upper |
|  | Age | -0.023 | 0.013 | 0.092 | 0.978 | 0.952 | 1.004 |
|  | Syndr | 0.968 | 0.460 | 0.035 | 2.632 | 1.069 | 6.480 |
|  | FH+ TAD | 0.962 | 0.452 | 0.033 | 2.617 | 1.079 | 6.346 |
|  | Constant | -1.700 | 0.636 | 0.008 | 0.183 |  |  |

Values in the table are unstandardized coefficient (B) with standard error (SE), p values,

Exp(B) estimated odds ratio and 95% confidence interval for Exp(B). Syndr: presence of syndromic features;

FH+ TAD: positive family history for TAD.
